# Individuals’ experiences in U.S. immigration detention during the early period of the COVID-19 pandemic: major challenges and public health implications

**DOI:** 10.1186/s40352-023-00211-2

**Published:** 2023-02-17

**Authors:** Caroline H. Lee, Nishant Uppal, Parsa Erfani, Raquel Sofia Sandoval, Kathryn Hampton, Ranit Mishori, Katherine R. Peeler

**Affiliations:** 1 Harvard Medical School, 25 Shattuck St, Boston, MA 02115 USA; 2 Brigham and Women’s Hospital, 75 Francis St, Boston, MA 02115 USA; 3 The Johns Hopkins Hospital, 1800 Orleans St, Baltimore, MD 21287 USA; 4 Physicians for Human Rights, 256 W 38th Street, 9th Floor, New York City, NY 10018 USA; 5 Georgetown University School of Medicine, 3900 Reservoir Road NW, Washington, DC 20007 USA; 6 Division of Medical Critical Care, Boston Children’s Hospital, 300 Longwood Avenue, Bader 634, Boston, MA 02115 USA

**Keywords:** Immigration detention, COVID-19, Immigrant health, Migrant health, Qualitative analysis

## Abstract

**Background:**

Individuals held in carceral settings were significantly impacted by the COVID-19 pandemic. However, limited research exists of the direct experiences of individuals detained by the United States (U.S.) Immigration and Customs Enforcement (ICE). This study illustrates the major challenges described by individuals held in ICE’s immigration detention centers during the initial spread of COVID-19.

**Methods:**

We interviewed 50 individuals who were released from ICE detention between March 15, 2020 until August 31, 2020. Participants were recruited through immigration attorneys. Responses to a semi-structured interview were documented. Quotes from these interviews were thematically analyzed.

**Results:**

Study participants were detained in 22 different ICE detention centers, which were located across 12 states, in both county (41%) and privately-contracted facilities (59%). The major themes that emerged from interviews included inadequate protections against COVID-19, denial of physical and mental healthcare, and experiences of retaliation in response to self-advocacy. These issues perpetuated emotions of fear, distrust, and helplessness in individuals in immigration detention centers.

**Conclusions:**

This study represents the largest analysis of experiences of ICE-detained immigrants during the early months of the COVID-19 pandemic. To ensure the rights to health and wellbeing for this population, further actions should include improving public health conditions, protecting against human rights violations, addressing barriers to healthcare access, ensuring transparency about conditions in detention centers, and moving toward decarceration.

**Supplementary Information:**

The online version contains supplementary material available at 10.1186/s40352-023-00211-2.

## Background

The use of administrative detention for relief-seeking immigrants has grown dramatically over the last several decades in the United States (U.S.). In 2001, the average daily population of detained immigrants in the U.S. was 19,000 (Detention Watch Network, 2021). This number steadily increased to over 33,000 in 2010 up to a peak of over 50,000 in 2019 (Detention Watch Network, 2021). Just prior to the onset of the COVID-19 pandemic in February 2020, more than 39,000 individuals were detained by Immigration and Customs Enforcement (ICE) in detention centers across the country (U.S. Immigrations and Customs Enforcement, 2021).

Even prior to the COVID-19 pandemic, there were notable reports by whistleblowers, journalists, grassroots organizations, policymakers, and the Department of Homeland Security (DHS)’s Office of the Inspector General about the substandard conditions in immigrant detention centers (Government Accountability Project, 2021; Samuels, 2018; Sergent et al., 2019; Department of Homeland Security Office of Inspector General, 2016). These reports liken these centers to prisons and jails, despite the civil nature of immigrant detention (Saadi et al., 2020). In particular, numerous health concerns have been reported, including: delayed and inadequate healthcare delivery, poor living conditions conducive to the spread of infectious disease, and environments placing children at risk of harm (Keller et al., 2003; Rubio, 2021; Lo et al., 2021; Peeler et al., 2020). These systemic issues have even played significant roles in the deaths of individuals held in ICE custody, resulting from suicides and either delayed or substandard medical care (Parmar et al., 2021; Erfani et al., 2021a).

The COVID-19 pandemic has impacted the population detained by ICE in consequential ways (Meyer et al., 2020; Tosh et al., 2021). Given the congregate nature of carceral settings, especially the high-density living conditions and the limited ability of detained individuals to implement preventive and self-protective measures, such individuals are at an increased risk of contracting COVID-19 (Lopez et al., 2021). In April 2020, ICE published its Enforcement and Removal Operations COVID-19 Pandemic Response Requirements (PRR) detailing how the organization was minimizing detained individuals’ risk of contracting the disease (U.S. Immigrations and Customs Enforcement, 2020). However, despite these guidelines, unannounced inspections of facilities subsequently revealed inconsistent mask wearing and insufficient SARS-CoV-2 testing, among other failures to uphold its policies (Department of Homeland Security Office of Inspector General, 2021). The SARS-CoV-2 case rates also remained significantly higher among detained individuals compared to the general U.S. population during the first six months of the pandemic (Erfani et al., 2021b; Casanova et al., 2021).

Previous research has largely relied on secondary sources, such as through interviews with facility staff, or focused on detention centers in specific geographic regions in the U.S. (Puthoopparambil et al., 2015; Durkin et al., 2020). Various reports from community organizations have also played an important role in real-time reporting of deaths, quarantine and transfer policies, and conditions inside immigration detention (Freedom for Immigrants, 2021; RAICES 2020). However, there is a paucity of in-depth research studies that detail the lived experiences of this large population through methodologically-collected information from the primary sources of individuals held in ICE custody.

We sought to understand the experiences of this population living in immigration detention centers during the early months of the COVID-19 pandemic as a means of informing the public health policies necessary to uphold their right to health.

## Methods

### Study design

A qualitative semi-structured interview study was conducted to understand the experiences of individuals detained in ICE facilities during the initial months of the COVID-19 pandemic. The interview included questions on the following topics: 1) Demographics; 2) COVID-19 Education; 3) Access to Sanitation & Hygiene Products; 4) Social Distancing, 4) Screening & Quarantine; 5) Punishment & Retaliation; and 6) Release from Detention designed to compare individuals' experiences to relevant PRR and National Detention Standards [see ‘Additional file 1’] (U.S. Immigrations and Customs Enforcement, 2020; U.S. Immigrations and Customs Enforcement, 2019). Responses to open-ended questions and additional quotations determined as salient by study staff were recorded and analyzed. The qualitative research framework of thematic analysis was used to generate themes from the interview data and was coded with team-based consensus strategies (Butina, 2015; Vaismoradi, 2016). This study was considered exempt from further review by the Harvard Medical School Institutional Review Board (IRB20-0915) and the Physicians for Human Rights’ Ethics Review Board.

### Participants and data collection

Study participants were recruited through immigration attorneys who had represented formerly-detained individuals, and who ascertained subject eligibility and interest through a pre-written script and recruitment flier. Both of the written recruitment information and verbal outreach process emphasized that declining to participate in this study would not impact their legal representation and relationship with their lawyer in any way, in order to minimize any pressure to participate. Inclusion criteria included age of 18 years or older, any gender, any country of origin and native language, location in the U.S. at the time of the interview, and status of being previously held in ICE detention with a release date on or after March 15, 2020. This date coincided with the week the U.S. issued its declaration of a national emergency due to COVID-19 (The White House, 2020).

Research team members (C.L., N.U., P.E., RS.S., D.G., K.P.) interviewed participants from July 13 to October 3, 2020. All interviewers were chosen due to their prior experiences working with individuals with vulnerable immigration statuses, including individuals seeking asylum or refugee-statuses, and underwent additional training to prepare for the sensitive information and potential disclosures of trauma reported by participants.

Interviews were conducted over the phone, mostly through WhatsApp, by research staff in either the participants’ native language (English or Spanish) or with an interpreter if needed. Interviews lasted one to two hours. Participants were offered a $40 gift card as reimbursement for phone minutes and a standard meal. All participants reviewed informed consent forms, both written in their native languages and also verbally with study staff, then provided formal verbal confirmation of their consent to participate in order to proceed with the questionnaire.

To protect the study participants’ identity, rigorous data protection policies were enacted and the minimum personal information from participants was collected. No names were requested by study staff, no audio was recorded, and no written consent was obtained to ensure anonymity and to protect participant safety. The study was designed so that no one could directly link any of the responses collected with a specific individual interviewed, and identifiable data was deleted as soon as possible.

Quotations were directly transcribed into the study management software REDCap (Research Electronic Data Capture) (Harris et al., 2009; Harris et al.; 2019). Spanish language responses were translated into English after the interview by native Spanish-speaking study staff (RS.S., D.G.). Given that recordings were not obtained to protect anonymity, full transcription of the interviews was not performed.

Throughout the study, careful consideration was enacted to ensure that the safety and health of all participants, through consultations with team members from Physicians for Human Rights with extensive experiences working with individuals in detention. For example, the survey was designed to minimize the potential risk for psychological distress and re-traumatization by emphasizing at multiple time points that participants could opt-out of answering any question, allowing individuals to respond with simple categorical answers instead of recounting on their experiences if desired, by the interviewer never explicitly asking participants to recount traumatic experiences, such as about violence or abuse, and only recording relevant information if it was spontaneously and voluntarily offered. All participants were also offered mental health resources from community organizations specialized in providing care for immigrants.

### Data analysis

Qualitative data was analyzed using thematic analysis, by developing a codebook using inductive and deductive approaches [see ‘Additional file 2’ and ‘Additional file 3'] (Thomas, 2006; Guest et al., 2012; Nowell et al., 2018). The codebook grouped each theme using an adapted socio-ecological model analyzing intrapersonal, interpersonal, and structural factors. This model has been previously used to qualitatively examine the experiences of vulnerable individuals in carceral settings (Alvidrez, 2019; White Hughto et al., 2018). This socio-ecological model was used to identify deficiencies in the public health protections provided inside immigration detention against COVID-19 largely on the structural-level, and then how staff and detained individuals responded on the interpersonal and intrapersonal-level.

Deductive codes were identified based on the questionnaire’s sections, specifically “living conditions”, “lack of access to care”, “inadequate resources”, “retaliation” and “release.” However, the subthemes in these sections and any additional themes did not emerge until the inductive coding process. To ensure trustworthiness, authors became familiarized with the primary data (C.H.L., N.U., P.E., RS.S., K.R.P.) (Harris et al., 2009). Coders (C.H.L., N.U., P.E., RS.S.) then independently coded a portion of the data (15%) and collaboratively and iteratively generated additional codes using consensus debriefing and researcher triangulation. Subsequent quotes (85%) were then independently coded by two coders each using the finalized codebook [see ‘Additional file 1’]. Codes were organized with the continuous use of peer debriefing. Qualitative data was uploaded, managed, and analyzed in QSR International’s NVivo (released in 2020) (QSR International, 2020).

## Results

### Participant demographics

A total of 62 individuals were referred by immigration attorneys to our study. 50 participants were successfully contacted, interested in participating, and interviewed (Table 1). Of these individuals, 76% identified as male and 24% as female, and the average age was 36.3 ± 8.3 years old (Standard Deviation). The participants came from 22 different countries of origin and spoke 11 unique languages, with English and Spanish being the 2 most frequently-spoken languages.

**Table 1:** Characteristics of Individuals (*N* = 50) and Represented Detention Centers (*N* = 22)

Characteristic	N (Number of Individuals Interviewed)	%
Gender		
Male	38	76%
Female	12	24%
Age Range		
20-29	10	20
30-39	23	46%
40-49	11	22%
50-59	6	12%
Country of Origin		
Mexico	9	18%
Venezuela	8	16%
El Salvador	4	8%
Cuba	3	6%
Uganda	3	6%
Other (n ≤ 2)	23^a^	46%
English-Speaking		
Yes	33	66%
No	17	34%
Length of Stay in Detention		
Average ± SD (days)	240.8 ± 173	-
Range (days)	14-732	-
Release Date (Month)		
March 2020	6	12%
April 2020	20	40%
May 2020	8	16%
June 2020	12	24%
July 2020	3	6%
August 2020	1	2%
Represented Detention Facilities		
Number of facilities that individuals were held in	22	-
Number of states that facilities were located in^b^	12	-
Detention Facility Management		
Public	9	41%
Private	13	59%
Core Civic	4	18%
GEO Group	4	18%
Others (Ahtna Inc, Akima, LaSalle Corrections)	5	23%

^a^Additional countries of origin: Barbados, Cameroon, Colombia, Ecuador, Eritrea, Ghana, Guatemala, Haiti, Honduras, Nicaragua, Russia, Rwanda, Sierra Leone, South Africa, Thailand, Trinidad and Tobago, Uzbekistan

^b^Detention facility locations by state: Arizona, California, Georgia, Louisiana, Massachusetts, New Jersey, New Hampshire, New York, Oklahoma, Pennsylvania, Texas, Washington

The individuals interviewed were detained at 22 different ICE detention facilities across 12 states (Table 1). Of the facilities represented, 41% were county-run, while 59% were privately-contracted facilities run by CoreCivic, GEO Group, Ahtna Support & Training Services, Akima Global Services, and LaSalle Corrections. Individuals were all held in adult facilities, except one who resided in a family residential center. Participants stayed in the detention facilities for an average length of stay of 241 ± 173 days (Standard Deviation). Eighty percent of the participants were released from their detention centers between April and June 2020.

### Thematic analysis findings

Thematic analysis of participant responses revealed various structural, interpersonal, and intrapersonal challenges faced during the initial months of the COVID-19 pandemic inside ICE detention centers. These themes are described below in two major areas: 1) Lack of resources to address individual’s concerns about COVID-19 management inside detention facilities, and 2) Barriers to self-advocacy regarding protections against COVID-19.

With respect to the first area, participants described a lack of resources inside detention facilities, including: 1) Crowded settings where detained individuals were unable to socially distance, 2) Limited to no availability of masks and soap, 3) Inconsistent COVID-19 testing availability and procedures, 4) Issues in healthcare access, and 5) Inadequate mental healthcare.

In the second area of concern, individuals reported barriers that made it difficult for them to self-advocate for their needs and request appropriate protections against COVID-19, including: 1) Failure of ICE staff to provide information about COVID-19, 2) Implementation of protective measures in response to public pressure or litigation, instead of in adherence to federal and local public health guidelines, and 3) Retaliation against individuals who requested improved conditions.

These areas of concern and related sub-findings are described below.

### Lack of resources during the early months of the COVID-19 pandemic

#### Inability to socially-distance

The physical environment placed detained individuals at a particularly high risk of contracting COVID-19. Congregate living settings were described as cramped and crowded, limiting detained individuals’ ability to practice social distancing during all periods of the day. The sleeping areas were commonly comprised of several bunk beds with many individuals in one room, which led to conditions that directly conflicted with official guidelines:

*"They also told us not to be in groups of 10 or more, but there were 50 of us in a bunker without a lot of space so we couldn't socially distance."* (Participant ID 45).

Individuals also faced challenges social distancing while in the dining halls, pharmacy lines, bathrooms, and recreation areas:

*"[There were] 60 people in one dorm, 60 in another. So 120 of us, we all shared one recreation area, maybe a 30x30 area with one basketball hoop.”* (Participant ID 41)

#### Lack of hygiene supplies

Participants described difficulties accessing basic protective equipment and materials such as masks, hygiene supplies, and cleaning materials to guard against COVID-19 transmission. Individuals described having insufficient access to cleaning supplies to sanitize the bathrooms, recreational areas, and commonly-touched items such as communal telephones. Additionally, interviewees described the rationing of soap and toilet paper, which were given out only on certain days of the week or month, instead of on an as-needed basis:

*“As far as hygiene - soap, shampoo, toothpaste - you get 1 a month. The hygiene kits are a tiny toothbrush, small white bar of soap, a 2.5-3 oz bottle of shampoo. That has to last you.”* (Participant ID 41)

When detention facilities lacked adequate supply of basic necessities, detained individuals were forced to rely on their personal money to purchase soap and hygiene products. Personal money was either received from outside support, such as through family members or friends, or directly earned through employment from the facility, which have historically provided wages below federal and state minimum wage standards (Thompson, 2012; Medina, 2021):

*"Around mid-April they [detention staff] started to bring soap, before that we would have weeks where we would not have soap. Sometimes they would forget to bring soap, we would request soap but they would ignore us. They never really cared. We would have to buy our own soap or use shampoo."* (Participant ID 14)

#### Inappropriate SARS-CoV-2 testing and isolation protocols

Detained individuals reported inconsistent SARS-CoV-2 testing or screening protocols due to multiple factors, including the misuse of solitary confinement in place of testing for symptomatic individuals and failures to respond to initial symptoms. As such, it was difficult to determine how many participants had contracted COVID-19 during their time in the detention facilities.

Several interviewees noted that newly-arrived detained individuals were rarely screened or quarantined for COVID-19, even if they presented with symptoms:

*"Another detainee had signs and was really sick with strong symptoms of COVID. She came in the end of March and developed these symptoms. I asked her if she was tested but she said she wasn't. They [detention staff] still let her in. She was sick, vomiting, diarrhea, feverish, and chest pains."* (Participant ID 14)

Detained individuals who reported symptoms of COVID-19 had difficulties getting tested and often did not undergo proper medical or isolation procedures. Furthermore, when detained individuals were tested, interviewees reported failures to subsequently disclose test results to the patient and provide appropriate medical care.

Individuals who reported symptoms consistent with COVID-19 could be placed in solitary confinement, without access to hygiene or medical care, which further discouraged people from reporting potential COVID-19 symptoms:

*"They [detention staff] decided who can see the doctor by the officers and nurse by walking around and seeing who looks really sick. They come with a stretcher and if you look really sick they take you on a stretcher. And if you were really sick they took you to “the hole” [solitary confinement]. Because they send you to “the hole”, some people don't say they are sick because they think “the hole” will make them worse. They keep it to themselves.”* (Participant ID 4)

#### Difficulties accessing medical care

Participants reported challenges accessing medical care due to lengthy wait times required to see clinical personnel and inadequate clinical staffing, which were further exacerbated by additional pressure on the medical system from COVID-19 cases. Interviewees confirmed that sign-up sheets were used to make appointments. However, detained individuals often had to place multiple requests or wait up to a week before being seen. During waiting periods, detained individuals reported that their symptoms worsened:

*"When you felt bad you would have to wake up at 5AM to sign yourself up on a list where then from there you would eventually get called to see the doctor. But sometimes that could take many days. It was only when you were really sick - almost dead - that they [detention staff] would actually take you to the doctors immediately.”* (Participant ID 32)

In some cases, detained individuals had to rely on outside legal counsel to obtain access to medical care, either directly from medical staff in detention or through referral to off-site facilities:

*"They [detention staff] weren't giving me medical care until I talked to my lawyer. The lawyer threatened to sue them. Then they took me to a medical center outside the detention center."* (Participant ID 20)

Even when being clinically evaluated, individuals reported being seen in facilities that primarily relied on nurses to evaluate patients, did not have necessary medications to treat acute illnesses, and/or did not provide appropriate follow-up care:

*"They [detention staff] just don't have a protocol for dealing with that pandemic. They only have the means to take an x-ray. There's nothing. They don't have enough medical personnel. There's only one doctor, and he doesn't treat you unless it's a serious illness, it's only the nurses that treat you."* (Participant ID 13)

#### Exacerbations of mental health challenges

Interviewees described significant mental health struggles during the COVID-19 pandemic. Individuals reported witnessing suicide attempts and other acts of self-harm coinciding with the beginning of the pandemic. Detained individuals reported experiencing verbal and physical abuse in detention that triggered memories of past trauma and exacerbated mental health conditions:

*“I was kidnapped and tortured back in my country. But here [in the US] it has been worse. Here I have been psychologically and emotionally tortured. Here they [detention center staff] play with us. All of the people still detained continue to suffer so much. The detention centers do not care about their detainees.”* (Participant ID 22)

Isolation measures used as precautions to limit the spread of COVID-19 further increased feelings of loneliness and worsened mental health challenges, as described by one interviewee:

*"They [detention staff] would make us stay in our cells for 23 hours at a time. You couldn't even shower. They would just give you food in the cell. We couldn't leave the room, watch TV, or make phone calls. It was difficult. It was hard to not talk to anyone."* (Participant ID 30)

### Limited self-advocacy to request protections against COVID-19

#### Concealment of information about COVID-19

Participants reported that detention staff did not provide enough education about COVID-19 protection strategies (masks, hand hygiene, etc.) and symptoms. Additionally, interviewees noted that detention staff actively concealed information about COVID-19 including the severity of the pandemic and the spread of COVID-19 within the facility:

*"They [detention staff] never gave us information or gave a talk about COVID-19 to us. They denied and denied that there were people in detention with COVID-19. But then army troops came to the center and set up tents in the yard to take care of sick people. When we saw that we knew that there were cases [of COVID-19] in the center."* (Participant ID 10)

Detained individuals who were exposed to SARS-CoV-2 were inconsistently quarantined and provided with testing results or follow-up medical care. Some interviewees even noted that medical staff, including nurses and doctors, were complicit in hiding information regarding the status of COVID-19 in the facility. Medical questions and complaints about symptoms consistent with COVID-19 were reportedly ignored by detention staff:

“*They [detention staff] didn't come to check my temperature until 3 to 4 hours after I was isolated. Eventually they came and told me they need to run some tests, but they didn't tell me what type of tests. I later found out it was a COVID test.”* (Participant ID 28)

There were also attempts to conceal the current conditions within ICE detention centers from news outlets and family members. One individual reported that communication with outside support, including phone calls and written letters, were monitored and blocked by staff. Another participant noted that ICE officials threatened to deport their friend if they spoke to the general public about the conditions inside the detention center after being released (Participant ID 39).

#### Changes largely enacted in response to outside pressures

Individuals described various mitigation strategies that were implemented during the early months of the COVID-19 pandemic, which likely reflected the implementation of recommendations by DHS and the Centers for Disease Control and Prevention (CDC). However, these changes were inconsistent across facilities and often in response to legal action, public protests, or COVID-19 outbreaks in facilities. For example, interviewees described that masks or medical care were only provided subsequent to legal actions:

*"I had severe headaches, sweats, felt weak, stomach pain, diarrhea. After I reported, I didn't see a nurse for one week. After I told my lawyer, they saw me after 2 hours."* (Participant ID 19)

Individuals also described that “pressure from outside” (Participant ID 41), such as from legal aid or news agencies, was pivotal to enacting protections within the facility. Interviewees underscored that improvements in their living conditions would often occur right before when members from the public entered the facility. These changes were perceived as purely for show and a strategy for the facility to continue the status quo:

*"They [detention staff] would only start to bring in supplies (soap, toilet papers) when people come in to take pictures. So it looks like we were lying."* (Participant ID 14)

Finally, participants also surmised that the increasing number of COVID-19 cases and deaths within ICE custody were important instigators of increased mask availability and improved quarantine and isolation standards in their facilities. As one individual noted:

*"After someone died in the detention center on ABC [news channel], they [detention staff] started giving us masks."* (Participant ID 4)

#### Retaliation in response to acts of self-advocacy

Individuals used various forms of self-advocacy in an effort to protect their rights and object to the perceived inadequate management of COVID-19. Various strategies included communicating grievances directly to facility staff, taking legal action, or going on hunger strikes. For example, participants described going on hunger strikes to advocate for the release of older individuals and those with chronic medical conditions who were at a higher risk of poor outcomes if infected with COVID-19. However, these actions would be met with threats and resistance from staff members, ranging from physical abuse to solitary confinement:

*“We went on [a hunger] strike for a week, and they [detention staff] were only threatening to take us to the coldest place in the world, where we can't communicate with anyone. They took some of our friends and told us that it is very dirty, very cold, they only give you a blanket, they don't turn off the light. So we decided to stop."* (Participant ID 13)

Immigrants reported that often staff responded to complaints with explicit acts of retaliation that further obstructed detained individuals’ abilities to continue to advocate for themselves and others. For example, participants reported that their attempts at communication with outside parties were stymied in the setting of increasing complaints:

*"They [detention staff] watch over everything you do (from the phone calls you make to what you write) and if they think that you're causing problems they punish you by taking you to a cell and isolating you where they do not give you enough food, you're isolated and you can't make phone calls to your family"* (Participant ID 10)

Other reported responses to protests included deprivation measures, such as revoking access to the commissary or to the showers, verbal threats, and even physical intimidation:

*"One colleague [detained individual] refused to eat despite being put in solitary confinement and he was gassed and pepper sprayed to make him eat."* (Participant ID 13)

Solitary confinement was also misused as a mechanism to punish protesters, prevent communication, and deter others from pursuing similar actions:

*"They [detention staff] removed inmates who they said were in charge of the strikes and sent them to “the hole" [solitary confinement] for 2-3 weeks, maybe longer. No one was in charge. It was just a tactic to separate people who could speak English and confront the officers."* (Participant ID 41)

## Discussion

To date, this is the largest-ever published study that analyzes the experiences of individuals who spent time in U.S. immigration detention during the COVID-19 pandemic. The themes identified from these interviews reveal major structural issues that led to improper management of COVID-19 in detention centers, including: lack of transparency, reactionary instead of preventative policies, crowded living conditions, lack of access to physical and mental healthcare, and inadequate protective materials against COVID-19. During this time, individuals additionally experienced largely negative interpersonal interactions with staff members, such as discrimination and retaliation in response to protest, which resulted in fear, distrust, and overall negative impacts on their mental health.

Our results suggest that many living conditions and healthcare systems inside detention facilities were in violation of the standards to control morbidity and mortality associated with COVID-19, which were set by DHS through the published PRR guidelines (Human Rights Watch, 2017; Department of Homeland Security Office of Inspector General,^ 2019^). For example, the PRR states that facilities must provide hygiene materials and masks, including “no-cost, unlimited access to supplies for hand cleansing” (U.S. Immigrations and Customs Enforcement, 2020). However, individuals instead reported that they experienced rationed and limited supplies and had to purchase these materials with their own personal money. Our data also suggests that there were additional violations of DHS guidelines for maintaining social distancing of at least 6 feet, educating individuals on about COVID-19 prevention, providing prompt medical care, and isolating and providing testing for COVID-19 for symptomatic individuals.

Our findings corroborate reports by the DHS’ Office of Inspector General that investigated major areas where “facilities struggled to properly manage the health and safety of detainees” during this same time period (Peeler et al., 2021; Department of Homeland Security Office of Inspector General, 2021, p.3). These investigations suggest that as COVID-19 continues to impact this country, changes are necessary to hold detention facilities accountable to official ICE and federal public health guidelines, protect the human rights of detained individuals, and explore alternatives to detention to prevent such abuses from occurring in the first place.

Our results revealed that individuals in U.S. immigration detention facilities during the early days of the COVID-19 pandemic lived in conditions direct violating international human rights standards, which uphold everyone’s rights to safe sanitation, access to healthcare, and freedom of expression (Rubio, 2021). The United Nations Universal Declaration of Human Rights includes “the right to a standard of living adequate for the health and well-being of himself” (Article 25) and “the right to freedom of opinion and expression” (Article 19) (United Nations, 1948). These specific articles were breached when individuals received inconsistent and inadequate protections against COVID-19, despite living in high-risk settings, in addition to the use of punitive and retaliatory measures in response to their concerns. These conditions increased individuals’ risk of contracting COVID-19 and impaired their abilities to self-advocate for their needs, thus creating a persistent environment of fear and mental suffering.

### Detention facilities failed to provide adequate protections against COVID-19

Before the development of vaccines and medications, CDC and other public health agencies encouraged all individuals to protect themselves by wearing masks, practicing social distancing, and using proper hygiene methods. However, the structural environment and failure of detention facilities to provide essential resources made these basic actions extremely difficult, if not impossible, for detained individuals to implement in comparison to the general U.S. population.

Interviewees reported crowded living conditions and inconsistent access to soap, cleaning supplies, and masks. There were also issues obtaining adequate healthcare treatment inside facilities, especially delayed COVID-19 testing and lengthy wait times to see medical professionals, until people were perceived as deserving of immediate care. Finally, the misuse of solitary confinement (as opposed to medical isolation) to isolate symptomatic individuals created fear of reporting potential COVID-19 symptoms, which may have propagated further viral transmission.

Given the lack of appropriate precautions against the spread of COVID-19 for individuals in ICE detention, improved public health measures are essential to ensuring the safety of this vulnerable population. For COVID-19 and other similar infectious diseases, the federal government must ensure facilities are provided with enough masks and hygiene materials, reduce the density of facilities through release of individuals, especially those at high risk of severe complications, and create designated quarantine and isolation measures that do not equate to solitary confinement, which carries significant negative physical and psychological impacts (James & Vanko, 2021). Additional mechanisms to improve the healthcare system in detention facilities include increasing staffing of qualified medical personnel, improving access to outside medical care, and increasing accountability for facilities in violation of official guidelines.

### Access to information & ability for self-advocacy are integral to public health

Various methods were used to control the knowledge detained individuals of accurate information about COVID-19 and suppress grassroots initiatives to advocate for improved public health protections. Interviewees also described staff members preventing the dissemination of detained individuals’ firsthand experiences inside the facility from reaching outside audiences, including family members and news outlets. Individual’s attempts to advocate for improved conditions were often met with disciplinary punishment including invasions of privacy, verbal threats, pepper spray, and solitary confinement. Inadequate access to health protections and harsh forms of retaliation eroded migrants’ trust in the detention staffs’ ability and commitment to reduce the spread of COVID-19. Thus, the abuse of trust by detention staff against migrants limited the effectiveness of public health interventions within detention facilities.

### Public health & policy implications

The results from this study indicate that immigration detention facilities failed to take basic public health measures and neglected to provide adequate medical care during the first six months of the pandemic. These reports, in tandem with the reported high rates of COVID-19 cases within detention centers, suggest that underlying systemic factors exacerbated by the pandemic contributed to these failures (Erfani et al., 2021b; Casanova et al., 2021). Interviewees also reported a mental health crisis caused by social and physical isolation, which reproduced conditions similar to solitary confinement, and the dismissal of mental health concerns by staff members and medical professionals. 

Further research and data are needed to understand the needs and experiences of this population. In particular, privately-contracted immigration detention facilities have been reported to have significant safety issues, understaffing, and substandard living conditions (Cho, 2021). While a majority of the detained individuals that were interviewed in this study were held in privately-owned facilities, we could not draw any conclusions about the differences in private versus publicly-run facilities in this specific study. Additionally, real-time data collection and transparency of symptomatic and confirmed COVID-19 cases among various facilities is essential for adequate public health monitoring and surveillance.

Substantial policy changes must be implemented to ensure that individuals in detention centers are not left in such vulnerable conditions in the future. During the early days of COVID-19, the individuals who were the highest risk for contracting an infectious disease were only provided with a paucity of masks, gloves, soap, and hand sanitizer. Instead, the government should prioritize resource allocation of protective equipment to ensure that adequate supplies are allocated for high-density settings, such as prisons, jails, and immigration detention centers. Additionally, the existing healthcare infrastructure inside detention must be equipped to care for patients, even during periods of increased demand, such as that was seen during the early waves of COVID-19. Our participants’ experiences in the healthcare system suggest a need for increased staffing of healthcare workers, improved protocols for transfer to outside hospitals for serious cases, and independent oversight of each facility’s medical system with a formalized certification process. In addition to physical health, the mental health crisis during the pandemic had drastic impacts on the population inside detention during this time. To safeguard their mental health, individuals need consistent access to mental health resources, such as counselors and psychiatrists, and protection of basic rights, such as access to showers and communication methods, even when strict isolation measures are in place.

Our interviews revealed that measures to protect the health of individuals in detention were commonly enacted in response to legal action and self-advocacy from the detained individuals themselves. Therefore, safeguarding individuals' ability to freely protest their conditions, communicate with outside resources while in detention, and acquire legal counsel are not only human rights, but essential tools to enhance public health and safety, especially in the context of a dangerous pandemic. In accordance, opportunities to report formal grievances should be protected, while banning retaliation for hunger strikes and other forms of self-advocacy. In particular, the use of pepper spray and tear gas in these settings may have worsened the spread and impact of COVID-19, as these chemicals have been correlated with increased respiratory illnesses and pathogenic spread (Stone, 2020).

ICE failed to protect the health and safety of individuals held in detention during the early months of the COVID-19 pandemic, as shown by the delayed response to provide basic hygiene supplies and masks, how it was impossible for individuals to socially distance themselves in facilities, and the healthcare system’s inability to promptly care for those in detention. Thus, the release of high-risk individuals and expanding alternatives to detention (ATD), such as parole, home visits, and GPS monitoring, represents important solutions to reduce the risk of transmission, disease, hospitalization, and death within such settings (Uppal et al., 2021). These policies have high rates of compliance, lower costs in comparison to detention, and represent a more humane treatment of immigrants (Noferi, 2015). Given the historic deficiencies and systemic inability of immigration detention facilities to appropriately care for the physical and mental health needs of detained migrants, decarceration of this administratively (non-criminally)-detained population represents a viable means to prevent transmission and to address the overburdened healthcare system in detention.

### Limitations

This study has several limitations. First, the study population was recruited through immigration attorneys. Due to possible selection bias, these experiences may be different from others in detention without access to legal counsel and represent individuals that attorneys may have felt would be either less traumatized by participating or who had significant experiences to report.

The Hawthorne effect, or observational bias, may have also played a role as interviewees may have felt expectations to recall the worst cases, even if those were not common (Sedgwick & Greenwood, 2015). Additionally, given the retrospective nature of the study, where the interviews occurred after participants’ stays in detention, the results may be hindered by recall bias. Despite these potential biases, the narratives still offer documentation of the harsh experiences of detention.

Additionally, since audio recordings were not taped due to concerns about confidentiality and safety, exact transcripts were not available and instead quotes were written down then analyzed if they were deemed as salient by the interviewee during the interview. This may result in confirmation bias on the part of the research team. However, whenever possible, efforts were made to involve multiple study staff in the interview process, so that one person was responsible of asking questions, while another individual was solely responsible for transcribing relevant quotes, in order to help ensure the accuracy of the quotes. No editing of these recorded quotes was performed, even for grammatical purposes, in order to accurately reflect the direct spoken words of the participants.

Finally, this study captures only a snapshot of the pandemic in the first six month when a shortage of masks and SARS-CoV-2 testing were pervasive throughout the U.S. Nevertheless, even at the time, public health guidelines addressing masking, social-distancing, hygiene measures, isolation and quarantine protocols were explicitly recommended by federal, state and local agencies. The limited timeframe of this study also means that this cohort represents individuals who were potentially affected by the original strain of SARS-CoV-2. Subsequent variants, such as Delta and Omicron, were found to be even more transmissible or severe, so the experiences of those in immigration detention during subsequent spreads of these variants may be completely different, and potentially even worse (Riediker et al., 2022).

Despite these limitations, and given the difficulty of accessing people in detention, these interviews represent a rich source of information with new findings to add to the existing literature.

## Conclusion

This study documents the major failures of U.S. immigration detention facilities to ensure safe conditions in detention in the early months of the COVID-19 pandemic. Major themes discussed by participants included difficulties protecting themselves against COVID-19 due to denial of basic and essential supplies, lack of implementation of known risk mitigation measures, failure to provide timely healthcare access, misuse of solitary confinement, and punishment in response to self-advocacy.

Overall, these findings are draw attention to the human rights abuses inside detention centers during the COVID-19 pandemic, and have important implications to the health of individuals in congregate carceral settings, including prisons and jails, both during and beyond the pandemic.

## Supplementary Information

**Additional file 1.** Interview Questionnaire.

**Additional file 2.** Codebook with Identified Themes and Subthemes.

**Additional file 3.** Treemap Representative of Themes’ Code Hierarchy: Relative sizes of themes correspond to the number of quotes coded under each theme.

## Data Availability

The datasets analyzed during the current study are not publicly available due to human subjects protections, but are available from the corresponding author upon reasonable request.
