# Supplementary material for: Individuals’ experiences in U.S. immigration detention during the early period of the COVID-19 pandemic: major challenges and public health implications
Source: Health Justice. 2023 Feb 17;11:8. doi: 10.1186/s40352-023-00211-2 (PMC9936455; doi:10.1186/s40352-023-00211-2)
Supplement: Supplementary file 1 — Additional file 1. Interview Questionnaire. [file 40352_2023_211_MOESM1_ESM.docx]

**Interview Questionnaire**

***COVDI-19 Education***

- Were there signs in the facility reminding you to practice good hand hygiene (i.e. regularly washing hands with soap and water for at least 20 seconds)?
- Were there signs in the facility reminding you of proper coughing etiquette (i.e. covering your mouth and nose with your elbow or tissue instead of with your hand)?
- Did the facility staff educate you about hand hygiene (such as washing your hands for 20 seconds) somehow other than signs?
- Did the facility staff educate you about coughing etiquette (such as coughing into your elbow or tissue) somehow other than signs?
- Were you told by facility staff to not touch your eyes, nose, or mouth without washing your hands?
- Were you told by facility staff to not share eating utensils, dishes, and cups?
- Were you told by facility staff to not shake hands, hug, or give high-fives to others?
- Were you told by facility staff to stay 6 feet / 2 m away from other people in the detention center as much as possible?
- Were you told by facility staff to avoid congregating in groups of 10 or more as much as possible?
- Were you told by facility staff about the specific symptoms of COVID-19?
- How did you first hear about COVID-19?

***Access to Sanitation and Hygiene Products***

- What did you use to clean your hands?
- How many times a day were you able to wash your hands with soap and water or hand sanitizer?
- Were you given an opportunity to clean your hands the majority of the time?
- How often did you have access to free soap and water and/or hand sanitizer when washing your hands?
- How often did you have access to free paper towels or hand drying machines for hand cleaning?
- Did you have access to no-touch trash cans to discard used paper towels after handwashing?
- Did you have access to hand sanitizer anywhere in the facility?
- How often did you have access to tissues for coughing or sneezing?
- Did you observe frequently touched surfaces in common areas (such as door knobs, light switches, countertops, recreation equipment) cleaned or disinfected?
- Did you observe frequently touched surfaces in the bathroom (such as toilets, toilet handles, sink faucets) cleaned or disinfected?
- Were you given a mask?

***Social Distancing***

- During your time in ICE detention how many other people slept in your room, not including you?
- How often were you able to maintain social distance (6 feet / 2 m away) in your sleeping area?
- How close was your nearest neighbor to you while sleeping?
- Were you told by facility staff to sleep “head-to-foot”?
- How often were you able to maintain social distance (6 feet / 2 m away) in communal eating areas?
- How often were you able to maintain social distance (6 feet / 2 m away) at lines for the pharmacy?
- How often were you able to maintain social distance (6 feet / 2 m away) at the communal showers?
- Did you have meals at the same time as people from other housing units?
- Did you have recreational time at the same time as people from other housing units?

***Screening & Quarantine***

- During your time in the ICE detention after March 15th, was your temperature taken?
- During your time in the ICE detention after March 15th, were you asked whether you had symptoms of COVID-19?
- Did you ever report having symptoms of COVID-19 or were you ever found to have a fever in the facility?
  - If yes: Were you seen by a medical professional? Where were you seen? How long did you have to wait to see a medical professional? Were you tested for coronavirus? Did you receive the result of your test? Was your test positive?
- Do you have any medical conditions?
- Were you asked whether you have these conditions at any point during your detention?

***Punishment & Retaliation***

- Did you report or protest any issues related to COVID-19?
- Did you face retaliation for your actions?

***Release from Detention***

- Upon your release, where did you go to live?
- How were you released?
- Were you given masks when released?
- Do you take medications for any medical conditions?
  - If yes: Did facility staff provide you with supply of your medications upon release (if so, how many days of medication were you provided?)
- Upon release, were you told by facility staff on what to do if you get sick in the community?
- Upon release, were you told by facility staff to isolate yourself by limiting contact with others in the 2 weeks after your release?
