# Supplementary material for: Individuals’ experiences in U.S. immigration detention during the early period of the COVID-19 pandemic: major challenges and public health implications
Source: Health Justice. 2023 Feb 17;11:8. doi: 10.1186/s40352-023-00211-2 (PMC9936455; doi:10.1186/s40352-023-00211-2)
Supplement: Supplementary file 2 — Additional file 2. Codebook with Identified Themes and Subthemes. [file 40352_2023_211_MOESM2_ESM.docx]

***Appendix A.*** Codebook with Identified Themes and Subthemes

|  | **Theme** | **Subthemes** | **Definition** |
| --- | --- | --- | --- |
| **Structural** | Concealment | - Of knowledge - Of conditions from public - Of responsibility | Misleading detained individuals or outside organizations by withholding or providing false information |
|  | Reactionary Changes | - To legal action - To audits - To protests - To COVID-19 Outbreaks | Changes only implemented in response to penalty actions or poor outcomes |
|  | Living Conditions | - Environment - Sleeping area - Abusive conditions - Dining - Labor - Use of personal money | Descriptions of conditions inside of the detention facility |
|  | Lack of Access to Care | - Wait times - Inadequate staffing - Medications - Mental health | Factors related to getting appropriate health care treatment |
|  | Inadequate Resources | - Hygiene products - Personal protective equipment - Cleaning supplies | Issues obtaining products to decrease risk of contracting COVID-19 |
|  | Retaliation | - Obstructed protest - Removal of privileges - Verbal retaliation - Physical retaliation - Solitary confinement - Threats | Responses to detainee actions with retaliative actions |
|  | Release | - Transportation - Quarantine - COVID-19 testing - Medical Care - Housing - Restricted freedom | Descriptions of conditions around release from detention facility |
| **Interpersonal** | Staff Interactions | - Verbal abuse - Physical abuse - Dismissal of concerns - Abuse of power - Supportive interaction | Descriptions of interactions with individual detention center staff members |
|  | Detainee Interactions | - Supportive interaction - Negative interaction | Descriptions of interactions with other detained individuals |
|  | Outside Support | - Family - Non-Profit - Friends - Government representatives - Lawyers | Support from individuals or organizations outside of the detention facility |
|  | Discrimination | - Race/Ethnicity - Limited English Proficiency - Gender & Sexual Minority - Mental health | Individual instances of discrimination based on personal identity |
|  | Protest Methods | - Legal action - Hunger strikes - Public outreach - Filing complaints | Descriptions of various protest methods detained individuals engaged in |
| **Intrapersonal** | Emotional Response | - Fear - Anger - Sadness - Distrust - Indignation | Statements conveying emotional experience of detention |
|  | Mental Health Concerns | - Suicides - Personal mental health condition - Trauma | Descriptions of personal or close experience to mental health concerns |
