# Supplementary material for: Individuals’ experiences in U.S. immigration detention during the early period of the COVID-19 pandemic: major challenges and public health implications
Source: Health Justice. 2023 Feb 17;11:8. doi: 10.1186/s40352-023-00211-2 (PMC9936455; doi:10.1186/s40352-023-00211-2)
Supplement: Supplementary file 3 — Additional file 3. Treemap Representative of Themes’ Code Hierarchy: Relative sizes of themes correspond to the number of quotes coded under each theme. [file 40352_2023_211_MOESM3_ESM.pdf]

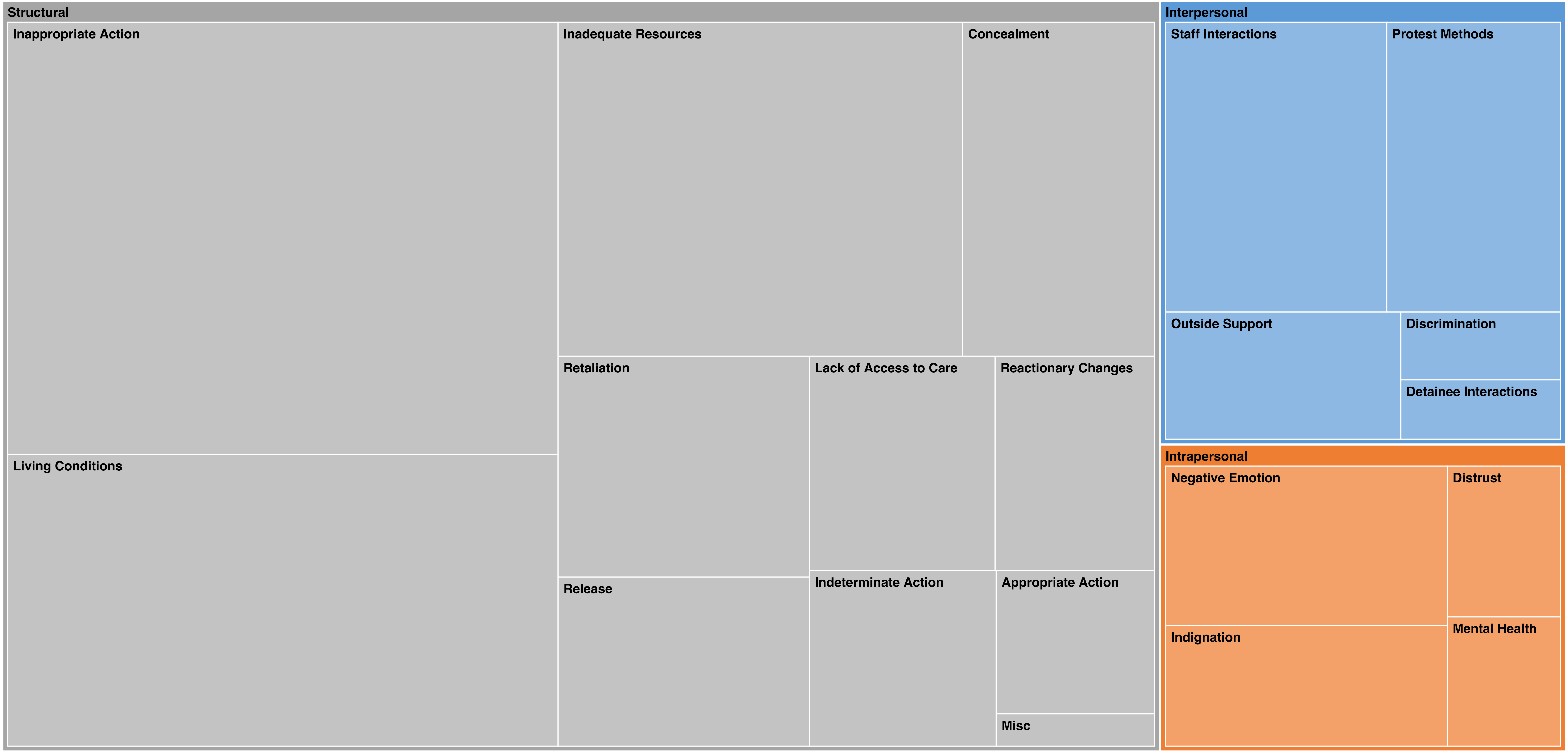
Structural
Inappropriate Action
Inadequate Resources
Concealment
Living Conditions
Retaliation
Lack of Access to Care
Reactionary Changes
Release
Indeterminate Action
Appropriate Action
Misc
Interpersonal
Staff Interactions
Protest Methods
Outside Support
Discrimination
Detainee Interactions
Intrapersonal
Negative Emotion
Distrust
Indignation
Mental Health
